# Supplementary material for: Effects of Adding Sphingomonas Z392 to Drinking Water on Growth Performance, Intestinal Histological Structure, and Microbial Community of Broiler Chickens
Source: Animals (Basel). 2024 Jun 28;14(13):1920. doi: 10.3390/ani14131920 (PMC11240382; doi:10.3390/ani14131920)
Supplement: Supplementary file 1 [file animals-14-01920-s001.zip › animals-3023955-supplementary.pdf]

Supplementary Materials

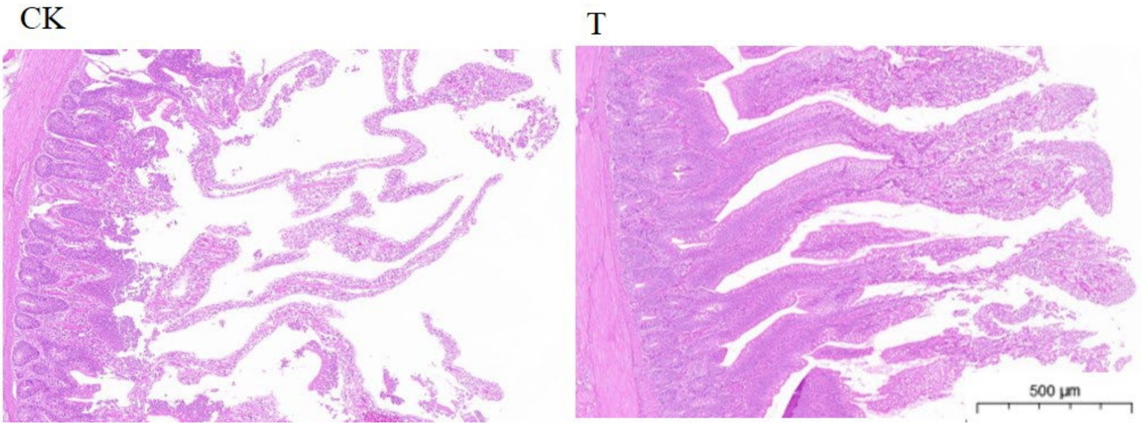

Figure S1. Histomorphology of duodenum of broiler chickens.
CK represents control group, T represents test group with Sphingomonas Z392 in drinking water.

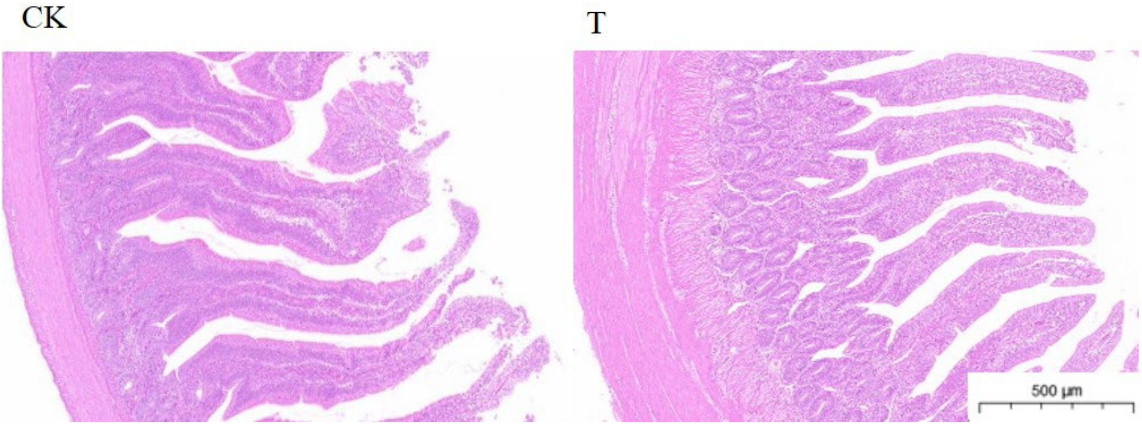

Figure S2. Histomorphology of jejunum of broiler chickens.

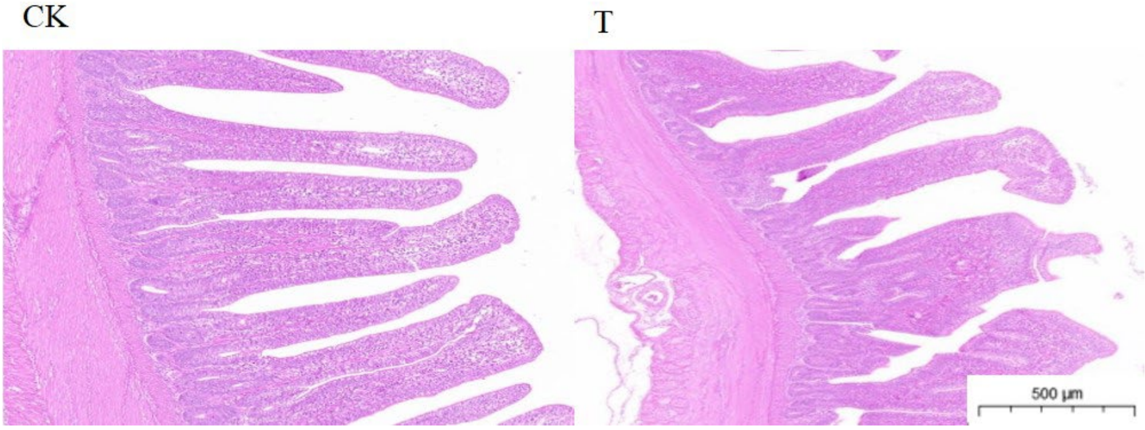

Figure S3. Histomorphology of ileum of broiler chickens.

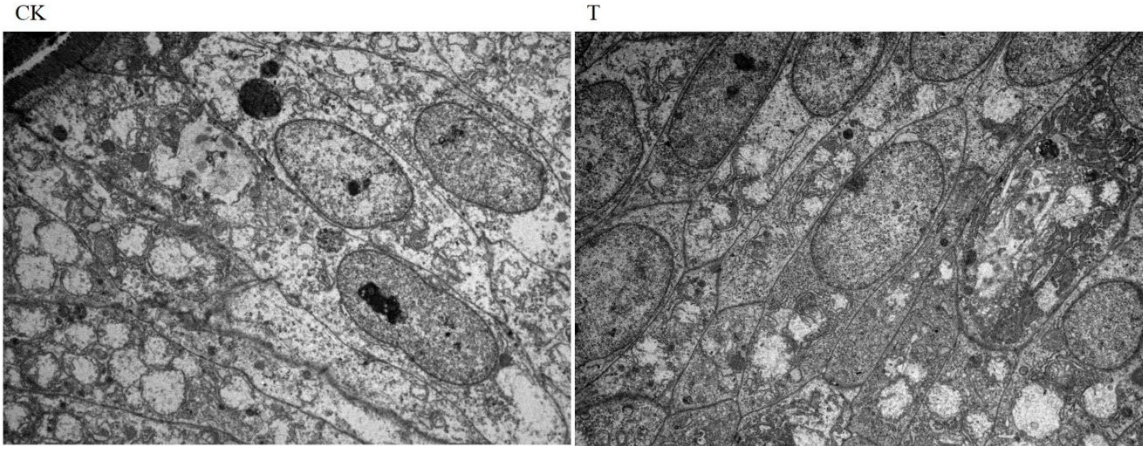

Figure S4. The villous epithelium of duodenum of broiler chickens (×5000).
CK represents control group, T represents test group with Sphingomonas Z392 in drinking water.

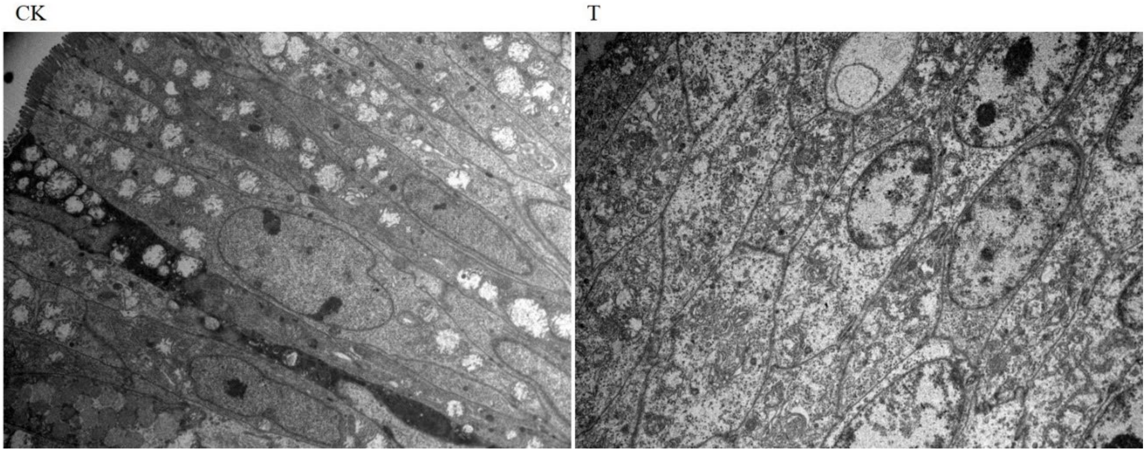

Figure S5. The villous epithelium of jejunum of broiler chickens (×5000).

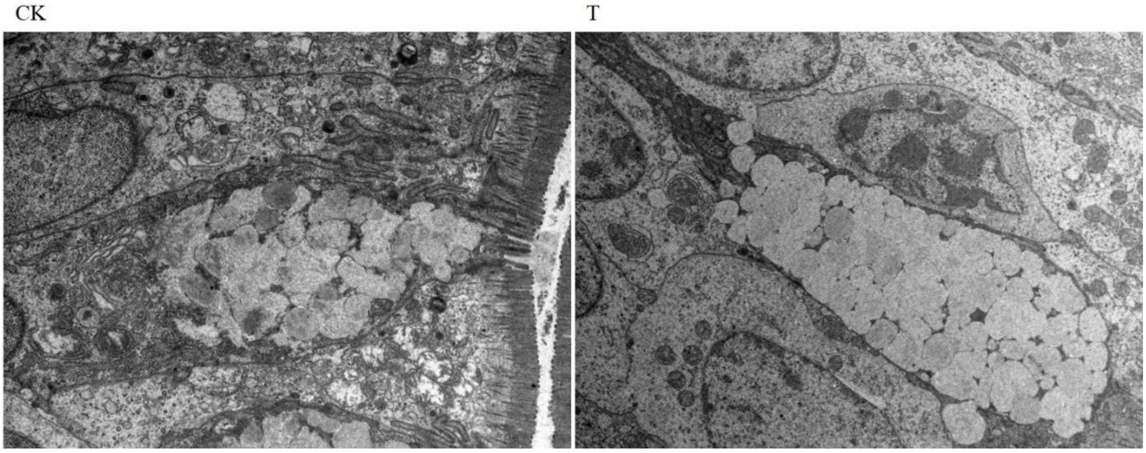

Figure S6. The villous epithelium of ileum of broiler chickens (×5000).

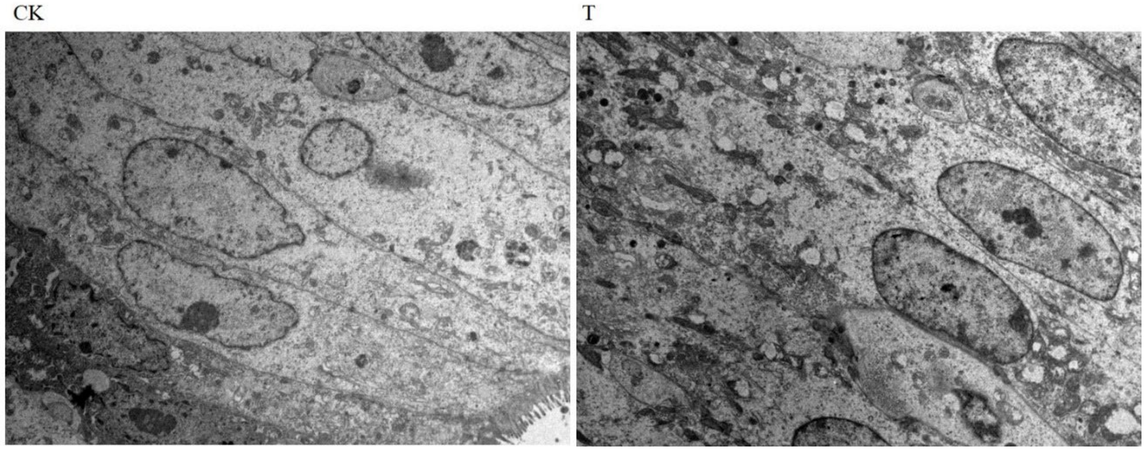

Figure S7. The villous epithelium of cecum of broiler chickens (×5000).

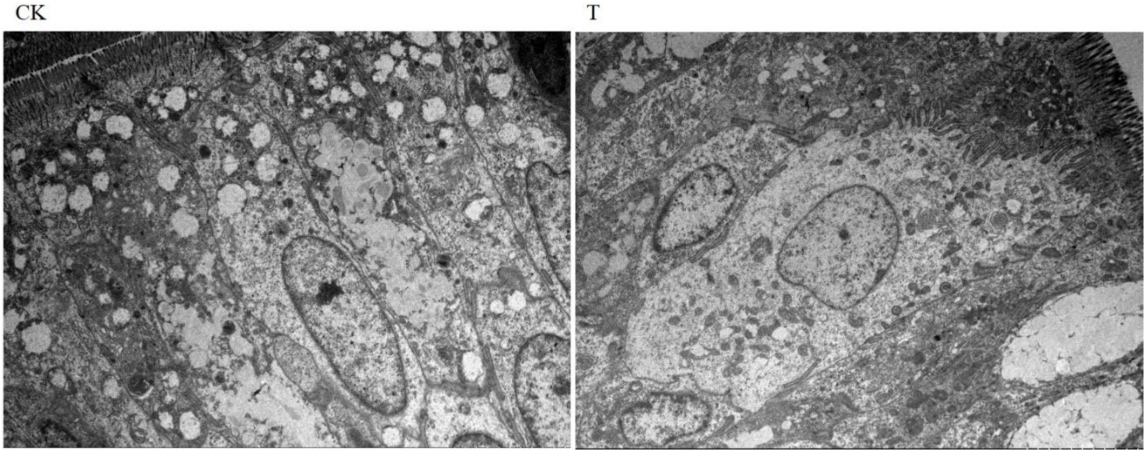

Figure S8. The villous epithelium of rectum of broiler chickens (×5000).
